# Clinical efficacy of etonogestrel implants on relieving dysmenorrhea in endometriosis and adenomyosis women for up to 3 years

**DOI:** 10.3389/fmed.2025.1460578

**Published:** 2025-03-21

**Authors:** Yinghua Li, Dingheng Li, Ting Feng, Chunfen Wang

**Affiliations:** ^1^Hangzhou Women’s Hospital, Hangzhou, China; ^2^The First People’s Hospital of Lin’an District, Hangzhou, China; ^3^Lin’an People’s Hospital Affiliated to Hangzhou Medical College, Hangzhou, China

**Keywords:** etonogestrel implants, endometriosis, adenomyosis, dysmenorrhea, women

## Abstract

**Background:**

Dysmenorrhea and menstrual disorders caused by endometriosis (EM) and adenomyosis (AM) have significantly affected the quality of life of a large number of women. As a highly effective clinical contraceptive measure, etonogestrel implants have been previously reported to relieve dysmenorrhea. However, the dysmenorrhea treatment and menstrual regulation effects of etonogestrel implants in AM and EM patients have not been systematically studied.

**Methods:**

This retrospective study followed up 100 patients with etonogestrel implants from May 2015 to October 2016, including 44 patients with EM and 56 patients with AM. The VAS scores of dysmenorrhea, menstrual volume, and related adverse events were measured at 12, 24, and 36 months after etonogestrel implantation in these patients.

**Results:**

In 100 EM and AM patients, dysmenorrhea significantly improved, with moderate and severe cases decreasing from 50 to 16 and 0% at 36 months. Amenorrhea increased over time, and frequent bleeding declined. Adverse reactions included weight gain (21%), acne (13%), and decreased sexual desire (10%). Serum CA125 levels dropped, confirming therapeutic efficacy.

**Conclusion:**

Etonogestrel implantation significantly alleviated dysmenorrhea symptoms in AM and EM patients.

## Introduction

The presence of endometriosis (EM) and adenomyosis (AM) affects 10 to 15% of the female population (1). Although AM and EM are not fatal, they are still the leading cause of pelvic pain and subfertility, and the leading cause of gynecological hospitalizations (2). AM and EM negatively affected women’s quality of life, work productivity, sexual relationships, and self-esteem (3). AM and EM not only cause pelvic pain, dyspareunia, amenorrhea, dysmenorrhea, and infertility, but also increase the risk of gynecologic malignancies (4, 5). Women with AM and EM can be asymptomatic, and some EM and AM lesions may heal on their own without diagnosis (6). Some patients with AM and EM can ultimately only be accurately diagnosed by laparoscopy, laparotomy, or hysterectomy, resulting in an immeasurable public health burden (7).

Painful symptoms play a critical role in AM and EM, serving not only as major clinical manifestations but also as key indicators for diagnosis and treatment evaluation (PMID: 34205040). Symptoms such as dysmenorrhea, chronic pelvic pain, dyspareunia, and cyclical or persistent abdominal pain significantly impact patients’ quality of life, work productivity, and psychological well-being (8). Dysmenorrhea, in particular, is one of the most common symptoms and often the primary reason for patients seeking medical attention (9). The occurrence of pain is primarily associated with abnormal uterine smooth muscle contractions, proliferation of nerve fibers, and localized inflammatory responses (10). Moreover, the severity of pain is often unrelated to the extent or depth of the lesions, making its management particularly challenging (11). Given the profound impact of pain on patients’ lives, investigating effective strategies to alleviate pain symptoms in AM and EM is of significant clinical importance, which is a central focus of this study on the efficacy of etonogestrel implants.

As an estrogen-dependent disease, AM (with or without EM) is sensitive to hormone-related drugs (12). Drug treatments for AM and EM include a range of options, such as oral contraceptive pills, oral progestin-only therapy, the levonorgestrel intrauterine system (Mirena), gestrinone, danazol, and gonadotropin-releasing hormone agonists (GnRH-*α*) (13). These therapies, however, are often associated with limitations, including long treatment durations, high recurrence rates after discontinuation, and various adverse effects, which can reduce patient tolerance and compliance (14). Etonogestrel implants have been widely used in clinical contraception with a 1-year unintended pregnancy rate of 0–0.5%. In clinical application, many patients with primary and secondary dysmenorrhea have been found to have significant relief of dysmenorrhea after placement of etonogestrel implants (15). Etonogestrel implants are very useful for patients with AM and EM who resist surgery or who still have unbearable menstrual cramps after surgery (16). Most patients with EM and AM still need contraception (17). Choosing a drug that is both contraceptive and relieving dysmenorrhea would be of greater benefit to the vast majority of women with EM and AM. In this study, we hypothesized that etonogestrel implants could effectively relieve dysmenorrhea in AM and EM patients. CA125 is a high-molecular-weight glycoprotein and a membrane antigen found on the surface of endometriotic lesion cells. Studies have shown that ectopic endometrial tissue has a robust ability to synthesize and secrete CA125, up to four times higher than normal endometrial tissue (18). Adenomyosis can lead to elevated serum CA125 levels due to secretion by ectopic endometrial tissue (19). Therefore, in this study, CA125 was used as an indicator for evaluating EM and AM, indirectly reflecting the activity and functional changes in EM and AM before and after different treatments. We hope our research will lead to further applications of etonogestrel implants in AM and EM therapy.

## Methods

### Study design

**Figure 1:**
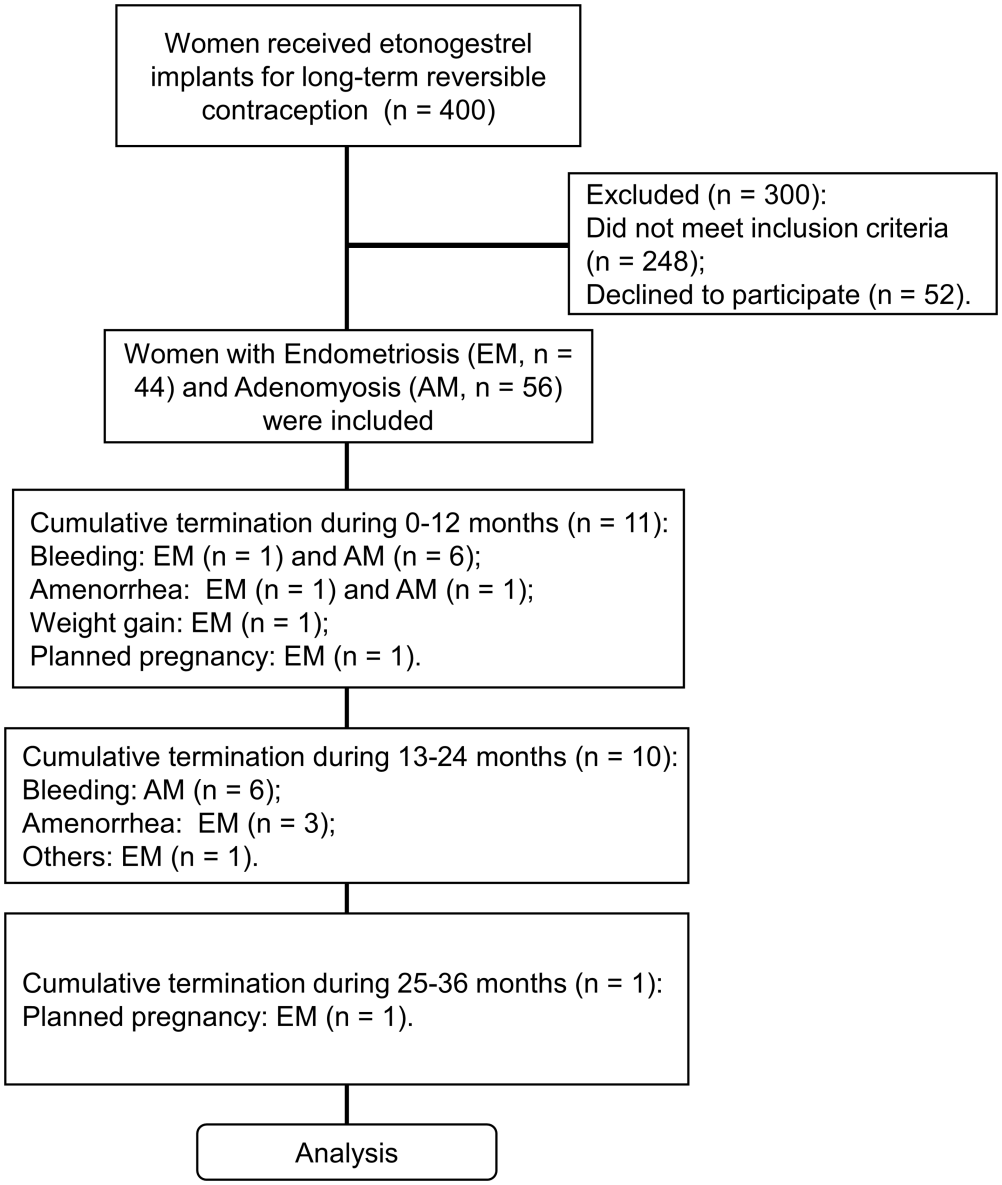
The flow chart diagram of this study.

This study is a follow-up observational study conducted from May 2015 to October 2016 on AM and EM patients who had etonogestrel implants placed. From May 2015 to October 2016, 400 contraceptive patients who requested contraception and were placed etonogestrel implants in Hangzhou Obstetrics and Gynecology Hospital and Hangzhou Lin’an District Maternal and Child Health Hospital outpatient clinic were analyzed. One hundred patients diagnosed with EM and AM by clinical symptoms, signs, transvaginal color Doppler ultrasonography, and serum CA125 levels were selected as the research subjects. There were 44 EM patients and 56 AM patients. 11 cases of etonogestrel implants were taken out after 12 months, and the continuation rate in 12 months was 89.0%. Between 13 and 24 months, an additional 10 patients had the implants removed. Therefore, a total of 21 removals occurred over the 0–24-month period, leading to a continuation rate of 79.0% at 24 months. One case was taken out in the third year. The main reasons for removal were bleeding or amenorrhea, weight gain, planning to become pregnant, etc. The relevant details and research process were shown in Figure 1.

### Diagnosis criteria

Patients in this study were diagnosed with AM or EM prior to the placement of etonogestrel implants, based on clinical symptoms, signs, transvaginal color Doppler ultrasonography, and serum CA125 levels. The diagnostic criteria followed the 2015 Guidelines for the Diagnosis and Treatment of Endometriosis issued by the Endometriosis Collaboration Group of the Obstetrics and Gynecology Branch of the Chinese Medical Association.

### Diagnostic criteria for EM

Clinical Symptoms and Signs: Pelvic pain, infertility, and menstrual irregularities. Imaging: Transvaginal ultrasound is valuable for diagnosing ovarian endometriotic cysts, typically presenting as anechoic regions with dense internal echoes. Transvaginal or rectal ultrasound, CT, and MRI are useful for identifying deep infiltrating lesions in the rectum or rectovaginal septum. Laparoscopy: The gold standard for diagnosis, allowing direct observation of lesion morphology. Examination should include detailed assessment of the pelvic cavity, particularly the uterosacral ligament and ovarian fossa. Histopathology confirming endometrial glands and stroma, along with inflammatory responses and fibrosis, is required for definitive diagnosis. Serum CA125: Elevated CA125 levels are more commonly associated with severe EM, significant pelvic inflammation, ruptured ovarian endometriotic cysts, or coexisting adenomyosis. It is not useful for early-stage EM diagnosis. Cystoscopy or Colonoscopy: Indicated for suspected bladder or intestinal EM to exclude malignancy, with biopsy confirmation rates of 10–15%.

### Diagnostic criteria for AM

Symptoms and Pelvic Examination: Suggestive findings include an enlarged uterus with a firm, irregular shape. Imaging: Ultrasound: Shows uterine enlargement and thickened myometrium, often more pronounced posteriorly. Echogenic spots or streaks within the lesion may be seen, with indistinct boundaries. MRI: Identifies low-signal-intensity lesions on T1-weighted images and high-signal-intensity lesions on T2-weighted images. The junctional zone thickness exceeding 12 mm is a key indicator. Serum CA125: Elevated in most cases. Pathology: Histopathological confirmation is definitive.

### Participants

Inclusion criteria: Patients who visited Hangzhou Obstetrics and Gynecology Hospital and Hangzhou Lin’an District Maternal and Child Health Hospital outpatient clinic; patients who were placed etonogestrel implants for contraception; patients were diagnosed as AM or EM before surgery based on clinical symptoms, signs, transvaginal color Doppler ultrasound, or serum CA125 level.

Exclusion criteria: patients with abnormal vaginal bleeding; patients diagnosed with malignant tumors of uterine origin or precancerous lesions through diagnostic curettage or cervical biopsy.

All patients were followed up 12 months, 24 months, and 36 months after placement of etonogestrel implants. Telephone, outpatient follow-up and other methods were used to follow up patients. Designated staff responsible for conducting follow-up reviews and recording data were responsible for reviewing and recording. These tasks include assessing the severity of dysmenorrhea, evaluating menstrual conditions (through menstrual card analysis), documenting other adverse reactions and reasons for implant removal, as well as measuring serum CA125 concentrations. The mentioned activities are part of the routine evaluations performed during patients’ regular follow-up visits.

### Etonogestrel implants

The etonogestrel implant used in this study (produced by Organon, the Netherlands, trade name Ebanon, contains 68 mg of etonogestrel, production batch number: 211587/294559) has an effective duration of 3 years. The patients underwent gynecological examination and breast examination before etonogestrel placement, and their blood routine, blood biochemistry, and coagulation function were normal. Cervical cytology examination was used to rule out contraindications to the placement of etonogestrel. The operating doctor explained to the patient in detail the possible adverse reactions and precautions after etonogestrel implantation. Patients signed informed consent. On the 1st to 5th day of menstruation, the etonogestrel implant was placed by a trained and qualified surgeon and the etonogestrel implant special placer was used during the placement operation.

### Dysmenorrhea score

Pain scores were recorded by the VAS pain scale during each patient’s menstrual period. 0 points for no pain, 1–3 points for mild pain, 4–6 points for moderate pain, and 7–10 points for severe pain.

### Menstrual bleeding

Patients were asked to assess changes in menstrual bleeding patterns between the two surveys at each survey. Amenorrhea: No bleeding. Infrequent bleeding: 1–2 episodes of bleeding and/or spotting, frequent bleeding: >5 bleeds, regular bleeding: 3–5 bleeds and/or spotting, prolonged bleeding: >14 days continuous bleeding and spotting, and spotting: spotting alone.

### Statistical analysis

Data analysis was performed with SPSS 20.0 statistical software. Normal distribution is expressed as mean ± standard deviation. Prior to conducting the comparative analysis, a Kolmogorov–Smirnov test was performed to evaluate the normality of the data distribution. The results demonstrated that the data did not conform to a normal distribution. Consequently, the Friedman test, followed by Dunn’s multiple comparisons test, was employed for the comparative analysis.

## Results

### Characteristics of the women with EM and AM

This study included 100 patients with preoperative diagnosis of EM and AM by clinical symptoms, signs, transvaginal color Doppler ultrasonography, and serum CA125 level detection. There were 44 EM patients and 56 AM patients. As shown in Table 1, the age of these patients was 20–45 years (33.81 ± 5.24), the pregnancies were 0–9 with an average of 2.8, and the parity was 0–3 with an average of 1.2. The patients were all married and had no reproductive requirements at present, and required contraception. Among the 100 study subjects, 34 patients had severe dysmenorrhea before placement, and needed to take painkillers or intramuscular analgesics for pain relief. There were 12 patients with AM who had been treated with Mirena before placement, and etonogestrel implants were placed after the device moved down or fell off.

**Table 1:** Characteristics of the women with endometriosis (EM, *n* = 44) and adenomyosis (AM, *n* = 56) who received etonogestrel implants for long-term reversible contraception.

Number of patients	100
Age at consent for implantation (years)	33.81 ± 5.24
BMI (kg/cm^2^)	24.73 ± 4.92
Gravidity (*n*, %)	
0	2 (2%)
1	9 (9%)
2	36 (36%)
≥ 3	53 (53%)
Parity (*n*, %)	
0	7 (7%)
1	46 (46%)
2	34 (34%)
3	13 (13%)
Marriage (*n*, %)	
Yes	100 (100%)
No	0 (0%)
Previous contraception methods	
No contraception control	14 (14%)
Intrauterine device	22 (22%)
Condom	43 (43%)
Oral contraceptive	9 (9%)
Mirena	12 (12%)

BMI: body mass index. Values were expressed as n (percentage, %) or mean ± SD.

### Alleviation of dysmenorrhea among women with EM and AM post etonogestrel implants

An analysis of the dysmenorrhea of the patients was presented in Table 2. Among the 100 patients, 81 had dysmenorrhea and 19 had no dysmenorrhea. The dysmenorrhea was significantly relieved 12 months, 24 months, and 36 months after etonogestrel placement. Sixteen (16%) patients had moderate dysmenorrhea and 34 (34%) patients had severe dysmenorrhea before implantation of the etonogestrel. The proportion of patients without dysmenorrhea at 12 months, 24 months, and 36 months after operation continued to increase, and the proportion of moderate to severe dysmenorrhea continued to decrease, and the difference was statistically significant compared with preoperative ones. These data suggested that etonogestrel implants significantly relieved or eliminated dysmenorrhea symptoms quickly and lastingly.

**Table 2:** Improvement of dysmenorrhea among women with endometriosis and adenomyosis who received etonogestrel implants for long-term reversible contraception.

Time point	No pain	Mild	Moderate	Severe	*p* value
Baseline (*n* = 100)	19 (19%)	16 (16%)	31 (31%)	34 (34%)	/
12 months (*n* = 89)	31 (34.8%)	20 (22.5%)	22 (24.7%)	16 (18.0%)	0.013^*^
24 months (*n* = 79)	37 (46.8%)	22 (27.8%)	17 (21.5%)	3 (3.8%)	0.022^#^
36 months (*n* = 78)	53 (67.9%)	16 (20.5%)	9 (11.5%)	0 (0%)	0.026^$^

Values were expressed as n (percentage, %). *p* value was derived from Chi-square test. ^*^Compared to baseline, ^#^compared to 12 months, ^$^compared to 24 months.

### Bleeding patterns among women with EM and AM post etonogestrel implants

The menstrual bleeding patterns of EM and AM patients at 12, 24, and 36 months after etonogestrel implants were analyzed and summarized in Table 3. Menstrual bleeding was significantly lower in patients 12 months after etonogestrel implants compared to baseline levels. Consistently, the number of amenorrhea patients with etonogestrel implants increased significantly after 24 and 36 months, and the number of patients with frequent or prolonged bleeding decreased significantly. These data demonstrated that etonogestrel implants could significantly reduce menstrual flow in AM and EM patients.

**Table 3:** Changes in bleeding patterns among women with endometriosis and adenomyosis who received etonogestrel implants for long-term reversible contraception.

Time point	Amenorrhea	Infrequent bleeding	Regular bleeding	Frequent bleeding	Prolonged bleeding	*p* value
Baseline (*n* = 100)	7 (7%)	12 (12%)	19 (19%)	29 (29%)	33 (33%)	
12 months (*n* = 89)	18 (20.2%)	21 (23.6%)	21 (23.6%)	20 (22.5%)	9 (10.1%)	0.000^*^
24 months (*n* = 79)	23 (29.1%)	29 (36.7%)	17 (21.5%)	8 (10.1%)	2 (2.5%)	0.023^#^
36 months (*n* = 78)	27 (34.6%)	32 (41.1%)	16 (20.5%)	3 (3.8%)	0 (0%)	0.312^$^

Values were expressed as n (percentage, %). p value was derived from Chi-square test. ^*^Compared to baseline, ^#^compared to 12 months, ^$^compared to 24 months.

### Adverse reactions in patients post etonogestrel implants

During the three-year follow-up period, the adverse reaction statistics of the patients were shown in Table 4. The main adverse reactions were weight gain in 21 cases (21%), acne in 13 cases (13.0%), breast tenderness in 9 cases (9%), abdominal pain in 6 cases (6%), mood changes in 4 cases (4%), sexual desire Decreased in 10 cases (10%), sleep disorder in 4 cases (4%), pigmentation in 2 cases (2.0%), etc. After placing etonogestrel implants in 100 patients with EM and AM, the dysmenorrhea of the patients was significantly relieved or even disappeared, and the menstrual flow was significantly reduced, suggesting that etonogestrel implants had a significant effect on the treatment of EM and AM.

**Table 4:** Adverse reactions among women with endometriosis and adenomyosis who received etonogestrel implants for long-term reversible contraception.

Number of patients	100
Weight gain	21 (21%)
Acen	13 (13%)
Breast pain	9 (9%)
Abdominal pain	6 (%)
Emotional lability	4 (4%)
Hypaphrodisia	10 (10%)
Sleep disorders	4 (4%)
Skin pigmentation	2 (2%)

Values were expressed as n (percentage, %).

### Changes in VAS scores and serum CA125 in AM and EM patients

**Figure 2:**
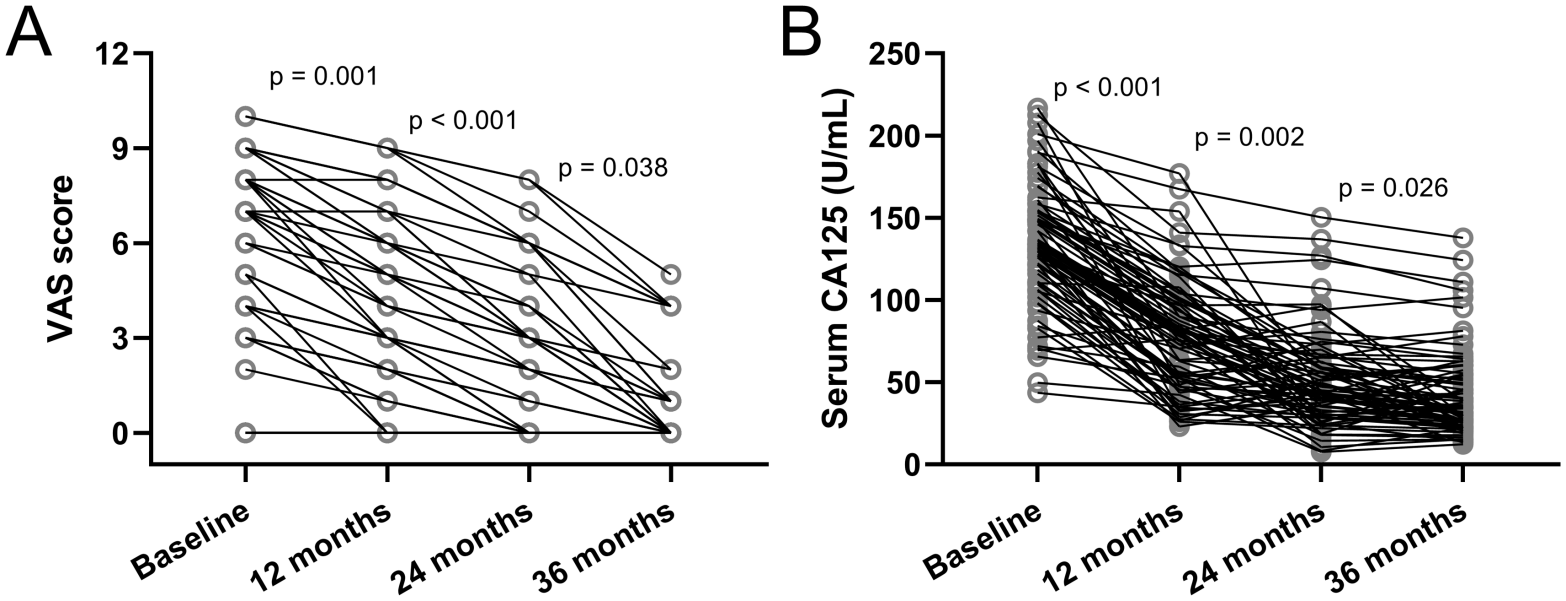
Changes in visual analogue scale (VAS) scores **(A)** and serum cancer antigen 125 (CA125) **(B)** among women with Endometriosis and Adenomyosis who received etonogestrel implants for long-term reversible contraception. *N* = 78. *p* values were acquired from Friedman test followed by Dunn’s multiple comparisons test.

VAS scores and changes in serum CA125 for all patients (*n* = 78) at the end of the three-year follow-up endpoint are shown in Figure 2. As shown in Figure 2A, the VAS of patients with dysmenorrhea decreases year by year, which further confirms that the patient’s dysmenorrhea was significantly relieved or even disappeared after placing etonogestrel implants in the previously reported data. In addition, the serum CA125 level of etonogestrel implants in AM and EM patients also gradually decreased, suggesting the therapeutic effect of etonogestrel implants in AM and EM (Figure 2B).

## Discussion

The purpose of this study was to investigate the clinical efficacy and adverse effects of etonogestrel implants for the relief of dysmenorrhea in patients with EM and AM (20). EM and AM are common diseases in obstetrics and gynecology, with an incidence of 2–48% (21). In recent years, the incidence of EM and AM has increased significantly. EM and AM are generally seen in women of reproductive age, and are more common in women aged 25–45. About 15–40% of AM patients have EM (22). Women with EM or AM are often accompanied by dysmenorrhea and increased menstrual bleeding, which seriously affects their quality of life and future fertility (23). The main causes of dysmenorrhea and miscarriage caused by AM and EM are endocrine dysfunction, decreased endometrial receptivity and immune factors (24). In addition, AM leads to impaired uterine spiral arterial remodeling and structural dysfunction of the uterine junction zone, which increases the reproductive risk in women (25). At present, hysterectomy is the accepted cure for AM. Although surgical treatment is straightforward, the subsequent loss of fertility, early perimenopausal symptoms, and pelvic floor dysfunction have a serious impact on the psychological and physical health of patients (26). Drug therapy for AM and EM mainly includes levonorgestrel intrauterine sustained-release system (Mirena), gestrinone, danazol, and gonadotropin-releasing hormone agonist (GnRH-*α*), etc. (27). However, EM and AM have a high recurrence rate after drug treatment.

Etonogestrel implants have been widely used in clinical contraception since it was approved by the U.S. Food and Drug Administration (FDA) in 2006 (28). The 1-year unintended pregnancy rate of etonogestrel implants is less than 0.5%, which is close to sterilization (29). In addition to the exact contraceptive effect, etonogestrel implants also have the advantages of high efficiency, good tolerance, fewer symptoms of estrogen deficiency, and quick recovery of fertility after removal (30). In clinical applications, etonogestrel implants have been found to be significantly relieved by etonogestrel implants in many patients with primary and secondary dysmenorrhea after placement, which is very valuable for patients with AM and EM who resist hysterectomy (31). Etonogestrel implants are thus a valuable supplemental treatment modality in some AM patients with EM who may still have unbearable dysmenorrhea after hysterectomy. The mechanism of action of etonogestrel implants for clinical contraception is the inhibition of ovulation. It can also induce endometrial atrophy, reduce menstrual flow, or even cause amenorrhea, and thus relieve dysmenorrhea (31). EM and AM are common and intractable diseases in women of reproductive age. At the same time, most patients with EM and AM still need contraception due to their poor intrauterine structure (32). Choosing a clinical strategy for both contraception and the treatment of dysmenorrhea will bring greater benefits to the majority of women with EM and AM.

This study followed up 100 patients with etonogestrel implants between May 2015 and October 2016, including 44 patients with EM and 56 patients with AM. We investigated the VAS score of dysmenorrhea, menstrual volume and related adverse reactions in patients with EM and AM after etonogestrel implantation at 12, 24, and 36 months. We found that in 19 patients with dysmenorrhea, dysmenorrhea was significantly relieved at 12, 24, and 36 months after the placement of etonogestrel implants. In addition, the statistics show that etonogestrel implants can significantly reduce menstrual bleeding. In this study, 22 patients had etonogestrel implants removed, of which 13 were removed due to vaginal bleeding, accounting for 59.09%. The main presentation in these patients was irregular bleeding and spotting after placement of etonogestrel implants. Vaginal spot bleeding is a very tricky problem in the use of etonogestrel implants (33). There is no proper solution yet, but the amount of bleeding is very small. Generally, no special treatment is required, and it does not affect daily life of the patients. Vaginal bleeding from etonogestrel implants is more likely to be accepted by patients after adequate counseling and explanation. Other adverse reactions of etonogestrel implants are mainly weight gain, acne, breast tenderness, mood changes, loss of libido, etc. However, these adverse reactions did not affect the continuation rate of etonogestrel implants. By analyzing the data in this study, we believe that etonogestrel implants are characterized by easy placement and long duration of treatment. The use of etonogestrel implants overcomes the characteristics of long-term oral tolerance or poor compliance of traditional dysmenorrhea drugs and frequent recurrence after drug withdrawal. Etonogestrel implants have few systemic adverse reactions and a good contraceptive effect. In addition, we also analyzed the CA125 levels in different time points of the two groups of patients. Since CA125 is abundantly present on the cell membrane surface of metaplastic epithelial tissues and can indirectly reflect the activity and function of ectopic endometrial tissue, it serves as a valuable indicator for observing EM and AM. It can indirectly reflect the effects of different treatments and pre- and post-treatment changes on the activity and function of EM and AM. Therefore, we believe that etonogestrel implants are a feasible way to treat dysmenorrhea in patients with EM and AM.

This study has several limitations. Its retrospective design introduces potential recall and selection biases, especially as 22 patients with incomplete data were excluded. The small sample size and lack of a control group limit the generalizability and comparability of the findings, while reliance on subjective outcome measures, such as VAS scores and self-reported bleeding patterns, may introduce reporting bias. Additionally, the study was conducted in a single center, which may reduce its broader applicability. Adverse reactions were assessed only over the three-year follow-up, leaving long-term safety and efficacy unaddressed. Serum CA125, used as a marker of disease activity, may have limited specificity, and the study did not evaluate the impact of etonogestrel implants on fertility, a key concern for many patients with EM and AM. Lastly, while the follow-up period was sufficient to observe medium-term effects, it does not provide insight into the long-term sustainability of the treatment benefits.

## Conclusion

In conclusion, we analyzed the therapeutic effect of etonogestrel implants on dysmenorrhea and irregular menstruation in AM and EM patients and its adverse effects in this study. We show that etonogestrel implants can significantly relieve dysmenorrhea and reduce menstrual bleeding in AM and EM patients. The main adverse effects of etonogestrel implants are irregular bleeding and spotting after placement, which are acceptable in most patients. We believe our study provides possible therapeutic options for the management of dysmenorrhea and menstrual irregularities in AM and EM patients with contraceptive needs.

## Data Availability

The original contributions presented in the study are included in the article/supplementary material, further inquiries can be directed to the corresponding author/s.
